# Reply to: “From derived signals to clinical stratification: interpreting EIT pulsatility in pulmonary embolism”

**DOI:** 10.1186/s13054-026-06343-9

**Published:** 2026-09-24

**Authors:** Shaofei Xu, Jiazheng Li, Ziqi Li, Yuxuan Cai, Junlai Zhao, Rongrong Zhu, Maokun Li, Weiwei Wu

**Affiliations:** 1 Department of Vascular Surgery, School of Clinical Medicine, Tsinghua Medicine, Beijing Tsinghua Chang Gung Hospital, Tsinghua University, No.168 Litang Road, Changping District, Beijing, 102218 China; 2 Department of Electronic Engineering, Tsinghua University, Beijing, 100084 China; 3 Department of Vascular Surgery, Chenzhou First People’s Hospital, The First Affiliated Hospital of Xiangnan University, Chenzhou, Hunan Province 423000 China

Dear Editor,

We thank Dr Chen for the thoughtful comments on our study of electrical impedance tomography (EIT)-derived pulsatility in pulmonary embolism (PE) [1, 2]. Dr. Chen raises important points regarding the relationship among the proposed indices and the interpretation of risk stratification. We appreciate the opportunity to clarify these issues.

First, we apologize for an inconsistency in the description of the statistical method in our original article. The combined model was described as using Z-score standardized variables with logistic regression, whereas the actual analysis used the unstandardized MI, DI, and SI values in an L2-regularized logistic regression model.The reported results of the combined model were obtained using this analytical procedure.We appreciate the opportunity to clarify this methodological discrepancy.

Second, we agree with Dr Chen that MI, DI, and SI are mathematically dependent, as MI=(1-DI) × (1-SI), which should be considered when interpreting their model coefficients.Nevertheless, the three indices have different physiological interpretations: MI reflects the overall spatial matching between ventilation and perfusion, whereas DI and SI reflect dead-space and shunt mismatch, respectively. Thus, although these indices are not statistically independent, they describe different aspects of ventilation–perfusion mismatch. We agree that the coefficient-based interpretation of their relative contributions should not be regarded as independent feature importance. The combined model was intended to assess overall diagnostic discrimination and provided little incremental discrimination beyond MI alone (AUC 0.820 vs. 0.817), as already described as marginal in our original study [2].

Finally, we agree that cross-sectional associations with established PE risk categories should be distinguished from prognostic prediction. Our study examined the association of EIT-derived indices with PE risk categories at the time of assessment and did not evaluate subsequent clinical outcomes. Accordingly, our findings demonstrate an association with established PE risk categories but do not establish prognostic value.

We thank Dr Chen again for highlighting these important issues.These clarifications better define the scope of our findings and the potential role of EIT as a non-invasive bedside tool for assessing PE-related pulmonary perfusion abnormalities. Future prospective studies are needed to further evaluate the clinical value of these EIT-derived indices.

## Data Availability

No datasets were generated or analysed during the current study.

## Abbreviations

EIT: Electrical impedance tomography
PE: Pulmonary embolism
MI: Matching Index
DI: Dead space index
SI: Shunt Index
AUC: Area under the receiver operating characteristic curve
